# Development and feasibility of a mobile phone application designed to support physically inactive employees to increase walking

**DOI:** 10.1186/s12911-021-01391-3

**Published:** 2021-01-21

**Authors:** Joanna Catherine Nicholas, Nikos Ntoumanis, Brendan John Smith, Eleanor Quested, Emmanuel Stamatakis, Cecilie Thøgersen-Ntoumani

**Affiliations:** 1 Physical Activity and Well-Being Research Group, School of Psychology, Curtin University, GPO Box U1987, Perth, WA 6845 Australia; 2 Western Australian Academy of Performing Arts, Edith Cowan University, Mount Lawley, Perth, WA 6050 Australia; 3 Charles Perkins Centre L6 West, Hub D17, School of Health Sciences, Faculty of Medicine and Health, University of Sydney, Sydney, NSW 2006 Australia

**Keywords:** Walking, Workplace, Physical activity, Behavior change, mHealth, Mobile apps, acceptability, Engagement, Perceived impact

## Abstract

**Background:**

Physical inactivity is a global health concern. mHealth interventions have become increasingly popular, but to date, principles of effective communication from Self-Determination Theory have not been integrated with behavior change techniques to optimize app effectiveness. We outline the development of the START app, an app combining SDT principles and 17 purposefully chosen BCTs to support inactive office employees to increase their walking during a 16-week randomized controlled trial. We also explored acceptability, engagement with, associations between app usage and behavioral engagement, and perceived impact of the app in supporting behavior change.

**Methods:**

Following development, fifty insufficiently physically active employees (*M* age = 44.21 ± 10.95 years; BMI = 29.02 ± 5.65) were provided access and instructions on use of the app. A mixed methods design was used to examine feasibility of the app, including the User Mobile App Rating Scale, app engagement data, step counts, and individual interviews. Linear mixed modeling and inductive thematic analysis were used to analyze quantitative and qualitative data, respectively.

**Results:**

Walkers rated the app quality favorably (*M* = 3.68 out of 5). Frequency of entering step counts (i.e., frequency of self-monitoring) on a weekly basis positively predicted weekly step counts measured via Fitbits at both the between-and within-individual levels. App features (entering daily step counts, reminders, and motivational messages) were perceived to assist walkers in fostering goal achievement by building competence and via self-monitoring.

**Conclusions:**

The START app may be a useful component of walking interventions designed to increase walking in the workplace. Apps designed to promote walking behavior may be effective if they target users’ competence and integrate BCTs.

*Trial Registration*: This study was part of a pilot larger randomized controlled trial, in which a component of the intervention involved the use of the mobile app. The trial was retrospectively registered with the Australian and New Zealand Clinical Trials Registry (ACTRN12618000807257) on 11 May 2018 https://www.anzctr.org.au/Trial/Registration/TrialReview.aspx?id=375049&isReview=true.

## Background

The high rates of physical inactivity among adult populations worldwide put many individuals at increased risk of a range of chronic diseases (e.g., cardio-metabolic diseases, many types of cancer, osteoporosis and dementia [1]) as well as premature mortality [2]. Adults in sedentary occupations (e.g. office workers) are sedentary for approximately 11 h per day [3], and are at greater risk of being overweight, obese, and physically inactive [3, 4].

### Mobile applications for the promotion of physical activity

The use of mobile applications (henceforth referred to as ‘apps’) for the promotion of physical activity has burgeoned in recent years. A systematic review of intervention studies (K = 27; 70% randomized-controlled trials; RCTs) revealed modest success of apps in increasing physical activity behaviors. The findings showed that apps were most effective when they were used for longer than 8 weeks in duration and when they formed part of a multi-component intervention [5]. Unfortunately, most commercially available apps are not evidence-based and have not been evaluated using scientific approaches [6]. Further, while the purpose of many apps is to increase physical activity behaviors, a review [7] showed that commercially available apps employed, on average, less than 4 behavior change techniques (BCTs) to increase physical activity. In terms of promoting walking, findings from a systematic review suggested that two BCTs, prompting self-monitoring of behavior and intention formation, may be perceived as most useful [8]. Another meta-analysis showed that prompting self-monitoring of behavioral outcomes and the use of follow-up prompts were the most effective BCTs in the prediction of physical activity maintenance in young and middle-ages adults [9]. When promoting physical activity and healthy eating in overweight and obese adults, a recent systematic review reported goal setting and self-monitoring of behavior as being the most effective BCTs [10]. Thus, it would appear prudent to incorporate a wider range of BCTs in apps designed to promote and sustain walking in insufficiently physically active adults. In addition to BCTs, in terms of specific app functionality, prompts and reminders (e.g., ‘push notifications’) have been found to promote app engagement and facilitate habit formation [11], including among office workers [12], and are, therefore, important to incorporate in app design.

### Interpersonal communication styles

Very few apps have used evidence-based principles of communication to promote BCT use and physical activity. Self-Determination Theory (SDT) [13] may be a useful theoretical framework to understand the effects of interpersonal communication. According to this theory [13], the communication style adopted by others (e.g., by exercise instructors or healthcare professionals), can be described as need-supportive or need-thwarting. A need-supportive style is characterized by features such as the provision of meaningful choice, competence-enhancing feedback, and demonstrating empathy or warmth. In contrast, someone who uses a predominantly need-thwarting style may offer little or no variety or choice, provide undermining feedback, or show no warmth or care towards the recipient. When a need-supportive style is adopted, the recipient is most likely to experience satisfaction of three basic human needs for autonomy, competence and relatedness, whereas a need-thwarting style will lead to the experience of frustration of these needs [14]. Evidence has shown that a need-supportive style will result in self-determined motivation, need satisfaction, sustained engagement, and psychological well-being [15, 16].

Although commercial apps may rely on, and contain features that align with, behaviour and motivational theory [17, 18], few studies have purposefully adopted a need-supportive communication style for delivery of content and BCTs within mobile applications [19]. There has been an increase in the number of studies investigating mechanisms and style of communication based on SDT used to deliver content via internet-based [20, 21] and via mobile technology (text messages [22]) with the aim of promoting physical activity. Results from several studies provide indicative evidence that need support delivered via agency-based means (i.e., not just face-to-face) has potential to lead to sustained physical activity behavior change [21, 22]. As such, it is important to ensure that the BCTs embedded within mobile apps are communicated in need supportive ways.

### App engagement and intervention efficacy

A systematic review found that app use was positively associated with increases in physical activity levels, although only three studies examined associations between app usage and changes in behavior [5]. Thus, it is evident that further studies are needed to examine the role of app usage in behavior change.

Systems usage data have served as the most commonly employed measure of engagement in mHealth interventions [23]. Such data capture immediate engagement with specific app features [24], however limit the ability to gauge in-depth engagement with the behavior change process (e.g., the extent to which participants have acquired new behavior change skills). Employing a range of methods has been advocated in order to capture both immediate engagement with app features and in-depth engagement with the behavior change process [23]. However, only few studies have combined a range of methods (i.e., app usage data, survey questions, and semi-structured interviews) to evaluate mHealth apps. In sum, the current study advances past literature by expanding the number of evidence-based BCTs incorporated in the app, adopting a need supportive communication style in the delivery of the content and BCTs, and using a mixed methods approach to tap into different levels of engagement with the app.

### Aims and hypotheses

The objective was to develop and examine the feasibility of START app among a sub-sample of participants taking part in a 16-week peer-led walking intervention designed to increase walking, improve health, well-being, and work outcomes in insufficiently active office workers. The aims were to (1) develop an app incorporating BCTs and need-supportive communication, including alpha and beta-testing the app to identify and rectify malfunctions prior to piloting with workplaces; (2) to examine the acceptability of the app among insufficiently active office workers; (3) examine engagement with the app (i.e., app usage) across the intervention period; (4) test whether weekly app usage was associated with weekly step counts retrieved via Fitbit devices provided to participants during the intervention; and (5) explore the perceived impact of the app in supporting behavior change by identifying specific features/components that were perceived by users as effective in supporting behavior change. In relation to the fourth aim, we hypothesized that use of the app would positively predict weekly step counts. It is expected that results from this study can be used to inform the development and evaluation of future apps designed to increase walking among insufficiently active overweight and obese office workers.

## Methods

### Research design

This study was part of a pilot RCT, in which a component of the intervention involved the use of the mobile app. The aim of START (Striding TowARds health and well-being Trial) was to test the effects of a 16-week workplace walking intervention on physical activity, health, well-being, and work outcomes [25]. This study adheres to the CONSORT guidelines and a CONSORT checklist is provided as Additional file 1. The study was conducted in Perth, Western Australia.

A two phase, mixed methods design was used to develop and examine the feasibility of the app, including surveys, individual interviews, objective step count data, and objective app usage data. Multi-phase approaches have been adopted in previous app development studies aimed at increasing physical activity among office workers [19]. Phase 1 (development) included app development, alpha-testing, and beta-testing. App development involved creating app content, incorporating evidence-based BCTs and need-supportive communication style into design features and content, and consulting with an external company to develop the app. Following initial development, members of the research team alpha-tested the app to identify preliminary malfunctions. The app was then beta-tested with a small group of participants, think-aloud interviews were conducted to identify malfunctions and to obtain further feedback to inform development prior to use in the main trial [26–28]. Phase 2 (feasibility) tested the acceptability (via a survey and semi-structured interviews), engagement with (via app usage data), associations between app use and behavioral engagement (via app usage data and step count data retrieved from Fitbit devices), and perceived impact of the app in changing walking behavior (questionnaires and interviews) within the context of a 16-week workplace walking intervention [23].

### Participants

For phase 1 (development), alpha-testing was completed by six members (*F* = 3, *M* = 3) of the research team. Members included behavioral scientists (*n* = 4), exercise and sport psychologist (*n* = 1), and exercise scientist (*n* = 1). Four participants (*F* = 1, *M* = 3) were recruited for beta-testing [27, 28]. Mean age was 37 years (*SD* = 15.4, range 23–63); all anecdotally reported that they had experience using physical activity app-based technology, met the recommended physical activity guidelines [29], and had completed tertiary education. Phase 2 (feasibility) included 50 walkers and 9 peer leaders from 5 organizations from the intervention arm of the RCT described earlier [25]. Walkers were organized into 9 groups (mean group size *n* = 5.0, *SD* = 1.63, range 3–7), each with a trained peer leader. Mean age of walkers was 44.21 years (*SD* = 10.95, range 24–66); mean body mass index (BMI) was 29.02 (*SD* = 5.65), the majority (84.0%) were female (*F* = 42; *M* = 8), and had an education level of diploma or higher (61.53%; Australian Qualifications Framework [30]). Eligibility criteria for the RCT required participants to be 18 + years, proficient in English language, have no medical or health problems that limited their ability to walk, and be able to walk continuously for > 15 min on a flat surface, being employed in a sedentary role (> 50% time sitting), and performing less than 150 min of MVPA per week (i.e., insufficiently active [29, 31]). The majority (66.6%) of peer leaders were female (*F* = 6; *M* = 3) and met recommended physical activity guidelines (77.7%) [29, 31].

### Procedures

All procedures performed were approved by Curtin University's Human Research Ethics Committee (HRE2017–0732). Written informed consent was obtained from all participants.

#### Phase 1: development

##### App features and theoretical framework

**Fig. 1:**
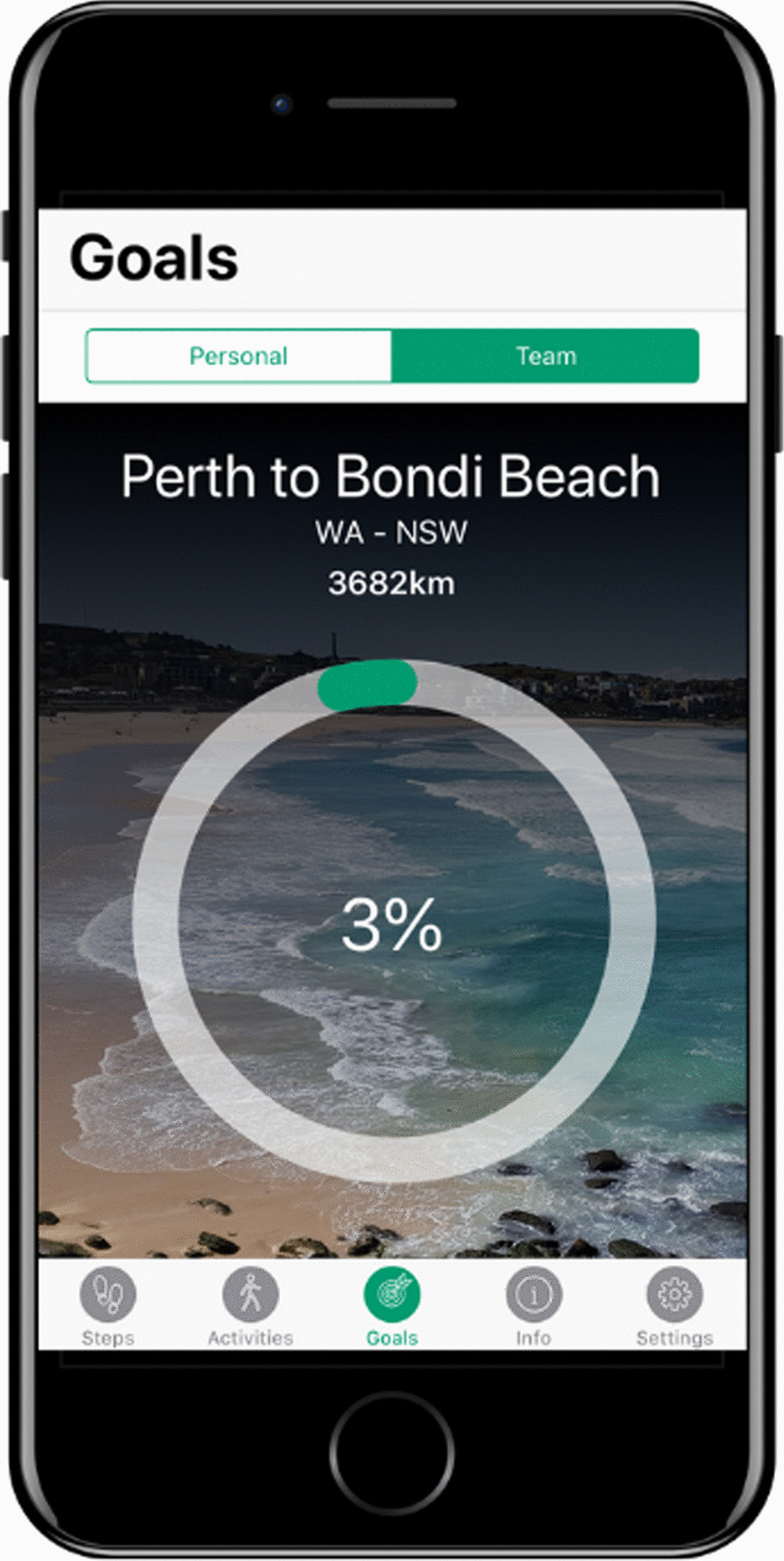
The START app. Image sources: app graphics developed by Reach Health Promotion Innovations and iPhone image sourced from Canva

A customized mobile application was developed for the iOS platform (Fig. 1). The START app integrated principles of need supportive communication with 17 purposefully chosen BCTs. These BCTs were chosen based on results of systematic reviews and meta-analyses assessing the efficacy of BCTs in promoting walking and general physical activity participation [8, 9], that have shown to be effective in promoting physical activity behavior in overweight and obese adults [10, 32], from a systematic review on app prompts and reminders to promote health behavior change [11], and from a review and content analysis of change techniques in popular commercial apps for weight management [33]. The BCTs were distributed across the static (constant) and dynamic (varied based on user interaction) contents of the app and are presented alongside corresponding app features in Table 1 [34]. Motivational messages and reminders within the app were designed to reflect a need-supportive style of communication [14] and are presented in Table 2.

**Table 1:** Static and dynamic START app features and corresponding BCTs [34]

Content	App feature	BCT
Static	Setting and adjusting goals, advice on overcoming anticipated barriers, information about planning activities	Goal setting behavior (1.1) Problem solving (1.2) Action planning (1.4)
	Information on the benefits of walking, injury prevention, frequently asked questions, and tips for making walks more interesting	Instruction on how to perform the behavior (4.1) Information about health consequences (5.1)
Dynamic	Encouragement to set and adjust goals	Goal setting behavior (1.1)
	Self-monitoring tools including request to enter daily step count and record structured walking activities	Self-monitoring of behavior (2.3)
	Weekly graph displaying progress towards goal	Feedback on behavior (2.2)
	Feedback on progress on achieving step goal delivered via messages using need-supportive communication (SDT)	Discrepancy between current behavior goal (1.6) Feedback on behavior (2.2)
	Request to set and adjust goals in light of progress	Review behavioral goals (1.5) Discrepancy between current behavior and goal (1.6)
	Plan weekly walks (when, where, with whom)	Action planning (1.4)
	Reminder messages linked to self-set plans using need-supportive communication (SDT)	Prompts/cues (7.1) Social reward (10.4)
	Request to rate feelings following structured walks, mid-walk motivational messages based on need-supportive communication (SDT)	Social support (practical) (3.2) Social support (emotional) (3.3) Monitoring of emotional consequences (5.4)
	Working with group members to achieve a team goal challenge (selected by the team). To achieve this, we pooled total step count for group members and displayed progress of mileage towards a well-known destination, based on group size and fitness level (e.g., walk from Perth to the Melbourne Cricket Ground)	Social support unspecified (3.1) Goal setting outcome (1.3) Graded tasks (8.7)

**Table 2:** Examples of motivational messages embedded in the start app based on need-supportive communication

Time within the intervention	Low weekly adherence (met steps goals 0–1 times)	High weekly adherence (met step goals 5 + times)
Early (weeks 1–3)	Good effort for reaching your step count goal once last week. There are many ways you can increase your step count—organized walks, active commuting, planned social walks with family and friends, finding nature walks, becoming more active in day to day life. Our app includes some ideas of how you can increase your walking on more days of the week to reach your goals	Wow, you really smashed it last week by exceeding your weekly goal. That's amazing and testament to your commitment. Think about what worked for you last week to achieve this great result and see if you can leverage from that again this week
Mid (weeks 9–10)	It can be helpful to reflect on why you joined the START program (e.g., health benefits, fitness, friendships). Trying to walk more on most days of the week will help you achieve what is important to you. What might you do the same and what might you do differently this week to meet your goal on more days?	Way to go last week! You exceeded the weekly goal. What helped you to achieve this success last week? Take a moment to enjoy the feeling and use that image if you ever find yourself struggling to meet your step count goals. Next week's challenge will be to meet your step count goal on 5 days per week
Late (weeks 13–16)	What opportunities might there be this week to increase your steps? You might find it helpful to plan ahead, considering what works for you, and also what makes it enjoyable for you. Thanks for joining us as part of the START program. We hope you will benefit from the changes you’ve made in the last 16 weeks and plan ways to sustain your new active lifestyle	Well done on meeting your goal on 5 days last week! Thanks for joining us as part of the START program. We hope you will benefit from the changes you’ve made in the last 16 weeks and plan ways to sustain your new active lifestyle

A main app feature included manual entry of daily step count. This feature was chosen as a means of implementing the BCT of self-monitoring [10, 34] and as it was not possible to integrate external step count data (e.g., from a Fitbit device) into the START app with the budget available for the app development. Another key feature was the team destination goal. Users could be allocated to a team, with each team able to select a destination to virtually walk to over the 16-week intervention. Virtual walks were categorized into easy, medium and hard difficulty level and based on group members individual baseline step counts, projected step count increases, and subjective walking pace and ability from initial group walks (calculations are provided as Additional file 2). Progress towards the destination was calculated automatically via the app by totaling daily step count entered into the app by the peer leader and walkers in each group. Two versions of the app were developed based on roles of users being either a walker or a peer leader. The main features available to walkers were enter daily step count, contribute to and view team progress towards destination goal, set reminders, set daily step count goals, and record walk activities. In addition to these features, peer leaders were able to record group attendance on group walks and set the team destination goal.

##### Alpha-testing

Members of the research team downloaded and trialed the START app. Team members tested all app features including setting a step count goal and entering daily step count and live walk activities for 10 days. Technical malfunctions and errors were reported to the corresponding author and app developers for rectification prior to beta-testing.

##### Beta-testing

Think-aloud walkthrough interviews are a common method for evaluating apps, and involve a user verbalizing experiences and perceptions as they navigate through an app [26, 27, 35]. Adopting protocols employed by White et al. [26–28], think-aloud walkthroughs were conducted to obtain preliminary user feedback, which was used to inform development of the final version of the app used in the main trial [19]. Participants were asked to navigate the app at their own pace and in the order they chose. Participants were asked to speak aloud to describe their actions and provide feedback while they navigated the app. If there were sections or interactive (i.e., starting a walking activity or recording steps) features of the app that participants missed, the researcher pointed them out to the participant and asked them to explore those features. Once the participant had explored all the features of the app they were asked to briefly summarize their perception of the functionality and aesthetics of the app. The researcher asked participants to elaborate on their think-aloud feedback and/or observable challenges they had whilst navigating the app. All interviews were conducted by the third author, a male sport and exercise psychologist with experience in conducting qualitative interviews.

#### Phase 2: feasibility

##### START trial

More detailed information about the START intervention procedures can be found in Thøgersen-Ntoumani et al. [25]. In brief, peer leaders and walkers attended training sessions which included details of the 16-week intervention and training in how to use the app. A phased approach was used whereby walkers were encouraged to participate in 2 peer-led walks per week and 1 self-organized walk a week for the first 10 weeks of the program. The number of peer-led walks reduced to 1 in weeks 7 to 10 and ceased in weeks 11 to 16, whilst the number of self-organized walks increased to 3 in weeks 7 to 10, and up to 5 in weeks 11 to 16.

Participants downloaded the START app onto a personal Apple iOS device; those who did not own an iOS device were provided an iPad for the duration of the intervention (*n* = 9). Walkers and peer leaders were provided a Fitbit Zip device and instructed to manually enter daily step count from the Fitbit into the START app at the end of each day. The walkers and peer leaders were advised that the research team were able to access their Fitbit step count data via an online platform.

##### User Mobile App Rating Scale (uMARS)

Following the trial, walkers (*n* = 34) and peer leaders (*n* = 7) completed an online questionnaire that included the User Mobile App Rating Scale (uMARS) [36]. Of the 18 participants (*n* = 16 walkers, *n* = 2 peer leaders) who did not complete the post-trial questionnaire, and therefore the uMARS, two cited changing workplaces, one perceived the program to be too long, one withdrew during the trial for unknown reasons, fourteen were uncontactable or did not report reasons for failing to complete the post-trial questionnaire. The Mobile App Rating Scale (MARS; developer version [37]) and uMARS (user version [36]) are frequently used tools to evaluate the quality of apps, including commercial apps for weight management [33]. The uMARS provides an overall app quality score and comprises four subcategories: engagement (entertainment, customization), functionality (ease of use, navigation), aesthetics (layout, graphics, visual appeal), and information (quality, quantity). We also assessed subjective app quality (whether users would recommend the app to others, whether they would pay for it) and perceived impact (questions pertaining to whether the app increased awareness of the importance of walking and assisted behavior change). Each item was rated on a scale of 1 to 5, with 1 being least desirable (e.g., *minimal/limited, not at all, or strongly disagree*) and 5 being most desirable (e.g., *intuitive/comprehensive, definitely, and strongly agree*). We also took into account qualitative feedback from participants who chose to provide a written response for the final question “*further comments about the app?”.*

##### Semi-structured interviews

Semi-structured 1-on-1 interviews were also conducted with walkers (*n* = 11) and peer leaders (*n* = 6) via phone or face-to-face at workplaces. All participants were invited to partake in an interview, however purposive sampling techniques were employed to ensure a range of participants (i.e., from all workplaces, low and high ratings for perceived impact of the app on walking behaviour, with varying step count, and with differing levels of engagement with the app) were interviewed. Interview guides were developed by the research team and included questions (provided as Additional file 2) pertaining to the acceptability and perceived impact of the START app (e.g., “Can you tell me about your experiences using the START app?”; “Did you choose some of the functions and not others?”; “What did you think about the weekly motivational messages? How did they make you feel?”). All interviews were conducted by the third author, a male sport and exercise psychologist with experience in conducting qualitative interviews. Participants were informed that their participation was voluntary and that they were able to stop at any time or decline answering questions. The average duration spent discussing the START app was between four and seven minutes. Data collection ceased once data saturation was met i.e., no new themes could be meaningfully generated, and there would have been no further value in interviewing more participants [38].

##### App usage

START app usage data was obtained from the hosting server and included *frequency* of entering daily step count (i.e., how often participants manually entered their daily total into the START app) and logging activities (e.g., entering information about walks such as when and with whom). This allowed for the computation of (1) the number of times on average participants recorded their step counts on the app across the intervention period, how often they met their walk goals, and how often each week they logged walk activities on the app, and (2) the percentage of participants who logged steps on at least a weekly basis throughout the 16-week intervention. Individual daily step count data were retrieved by the researchers via the Fitbit Wellness Platform after participants consented to providing us with access to the data.

### Analyses

Recordings of the think-aloud walkthroughs and post-trial interviews were transcribed verbatim and imported into NVivo qualitative data analysis software (Version 11). An inductive thematic approach was used to identify themes from think-aloud walkthroughs, and to measure acceptability and perceived impact of the app from post-trial interviews [39]. The first and third authors independently coded text to identify ‘meaning units’ regarding participants’ perceptions of the app. Meaning units that shared similarity in content were categorized into sub-themes and themes. A ‘critical friends’ approach was used with remaining authors to identify and challenge any weakness in the interpretation of meaning units and/or theme allocation, and to allow for exploration of alternative interpretations [40]. As a result, some themes and sub-themes were collapsed or removed, and some meaning units reallocated or removed.

All survey and app usage data were analyzed in IBM SPSS (Version 25.0). Linear mixed modeling was employed to examine how weekly app usage (daily step entering on the app, as well as logged walking activities) predicted weekly step count (*N* = 50). Both level 2 (between-person) and level 1 (within-person) effects were added to each of the two usage models; the level 1 variables were group-mean centered.

## Results

### Phase 1: development

#### Alpha-testing

Issues reported by the research team over 10 days of app use included: incorrect dates displayed in step count tab, incorrect numbers displayed for activity difficulty and feeling labels, starting a new activity causing the app to crash, opening the app from the daily step reminder notification causing the app to crash, and several missing hyperlinks in the frequently asked questions tab. All reported issues were fixed prior to beta-testing.

#### Beta-testing

Three themes emerged from the think-aloud interviews, namely, functionality, aesthetics, and information. Despite some of them reporting operational malfunctions (such as inoperable buttons), overall, participants perceived that the app was easy to use. Suggestions were made for improving aesthetics and information within the app to increase engagement, such as increasing font size and using bullet points, rather than presenting information in paragraphs. Decisions regarding changes to the app were based on feasibility and cost of implementing the changes. For example, in the step count function one of the participants indicated that they can more easily conceptualize the distance they walk in kilometers rather than steps so we included the following note; *There are approximately 1,300 steps in a km, based on an average stride length of 0.76 m.* Another example was regarding the team goals visual. One participant suggested including in the visual their individual percentage contribution to the team. This was not feasible due to time constraints as the teams would need to be formed first then the information would need to be fed back to the developer. Feasible recommendations were rectified, along with reported malfunctions, prior to the main trial.

### Phase 2: feasibility

#### Acceptability of START app

Walkers rated the app favorably: overall quality *M* = 3.68, *SD* = 0.44; engagement *M* = 2.95, *SD* = 0.59; functionality *M* = 3.86, *SD* = 0.52; aesthetics *M* = 3.55, *SD* = 0.60; and information *M* = 4.12, *SD* = 0.53. More detailed uMARS results are presented in Table 3. Peer leaders rated the app quality favorably (*M* = 3.89; *SD* = 0.39; Table 3). The main function used by peer leaders was entering group walks, which included recording walker attendance. Functionality (*M* = 4.25; *SD* = 0.46) and aesthetics (*M* = 3.86; *SD* = 0.33) were also rated favorably by peer leaders.

**Table 3:** Post-trial uMARS results for walkers (n = 34) and Peer Leaders (n = 7)

Subscale / item	Scale anchor 1	Scale anchor 5	Walkers				Peer Leaders			
			Mean	SD	Min score	Max score	Mean	SD	Min score	Max score
Overall app quality			3.68	.44	2.93	4.63	3.89	.39	3.21	4.33
Engagement	Not interesting at all ^a^	Very interesting; would engage user in repeat use ^a^	2.95	.59	1.60	4.20	3.34	.37	2.80	3.80
Functionality	No logical connection between screens at all/navigation is difficult ^a^	Perfectly logical, easy, clear and intuitive screen flow throughout, and/or has shortcuts ^a^	3.86	.52	3.00	5.00	4.25	.46	3.50	4.75
Aesthetics	Very bad design, cluttered, some options impossible to select, locate, see or read ^a^	Professional, simple, clear, orderly, logically organized ^a^	3.55	.60	3.25	5.00	3.86	.33	3.33	4.33
Information	Suspicious source ^a^	Definitely comes from a legitimate source ^a^	4.12	.53	3.25	5.00	4.11	.63	3.00	5.00
Subjective quality			2.46	.72	1.00	4.00	3.29	.53	2.25	3.75
Recommend app to others	Not at all	Definitely	2.65	.85	1.00	4.00	3.43	1.62	1.00	5.00
Times you would use app in next 12 months	None	> 50	2.94	1.50	1.00	5.00	4.50	.84	3.00	5.00
Would you pay for this app	Definitely not	Definitely yes	1.24	.61	1.00	3.00	1.71	.76	1.00	3.00
Overall star rating	One of the worst apps I’ve used	One of the best apps I’ve used	3.00	.78	1.00	4.00	3.43	.79	3.00	3.00
Perceived impact			2.92	1.16	1.00	5.00	3.23	1.10	1.00	4.33
Increase awareness	Strongly disagree	Strongly agree	3.15	1.31	1.00	5.00	3.29	1.25	1.00	5.00
Increase knowledge	Strongly disagree	Strongly agree	2.94	1.25	1.00	5.00	3.00	1.16	1.00	5.00
Changed attitude	Strongly disagree	Strongly agree	2.97	1.36	1.00	5.00	3.14	1.22	1.00	5.00
Increase motivation	Strongly disagree	Strongly agree	3.06	1.03	1.00	5.00	3.71	1.50	1.00	5.00
Encourage to seek further help	Strongly disagree	Strongly agree	2.59	1.13	1.00	5.00	2.71	.95	1.00	4.00
Increase walking	Strongly disagree	Strongly agree	2.82	1.36	1.00	5.00	3.57	1.40	1.00	5.00

^a^Example of an item within this subscale

Thematic analyses of interview data revealed that walkers’ and peer leaders’ perceptions of app acceptability aligned with uMARS ratings. Extracts from the interviews are presented in Table 4 for brevity reasons and a comprehensive table with additional quotes is provided as Additional file 2: Table S1. In terms of functionality, most walkers reported that the app was easy to use. Those who did not own an iPhone were supplied with an iPad (without a network subscription), which limited the use of some app functions, such as entering activities and receiving mid-activity motivational messages, due to the inconvenience of carrying a larger device and inability to connect to an internet source whilst walking. Although some walkers enjoyed entering daily steps, other walkers would have preferred the Fitbit to sync automatically and transfer step count to the START app. Several walkers who rated the app below midpoint on subjective quality and perceived impact reported (via written comments in the uMARS) engaging more with the Fitbit app than the START app. In terms of aesthetics, some walkers reported the app to be quite plain. Although the majority of walkers felt positively about the app overall, a small number reported that they prefer not to use technology or apps. The majority of peer leaders’ comments related to functionality and aesthetics, most stating that the app was easy to use and generally aesthetically pleasing (Table 4 and Table S1).

**Table 4:** Acceptability and perceived impact of the START app in supporting behavior change

	Theme	Sub-theme	Exemplar meaning unit
Acceptability	Functionality	Ease of use	Walker 1: “I found it pretty easy to use. I never had any problems with it. It was pretty intuitive and—yeah, it was pretty basic and effective, easy to read and understand.” Walker 5: “I actually found it very easy to work, to use, and—yeah, I thought it was just really basic and really just—I mean, it did what it had to do and easy to use.” Peer leader 3: “Yep. I used it for every walk and I found it really good, a really good way of recording the walks and it worked really well, so I would just insert the basic details, who’s going on the walk, what type of walk, and then go press start and stop. We pretty much used it all the time.”
		Manually entering daily step count and influence of Fitbit app	Walker 3: “I would only just say you could sync it in [with the FitBit]. That would be the only thing ‘cause we just live in a world where—we’re just so fast. Everything’s done for us. A bit lazy, I know.” Walker 11: “I love the little [START] app that you can enter your steps.” Walker 10: “I found the app to be really just an entry portal for data for the purpose of visibility for the Curtin START team. Predominantly I used the Fitbit app as the main source, and then just entered the step data into the START app.” Walker 14: “We needed to engage with the Fitbit app to interact with the START App, which made the START app redundant to the Fitbit app. If the Fitbit app automatically sent steps into the app, it would have been easier to engage with the app.”
		Limited to Apple devices	Walker 9: “… if it was designed for both Android and Apple, you’d be extremely successful at it.” Walker 14: “I also had to use an iPad as I did not have an iPhone making the app more inconvenient as I needed to be connected to wifi, which I did not have access to at work.”
	Aesthetics		Walker 6: “Yeah. Yeah, it was fine.” Walker 9: “It’s a bit plain, to be honest, aesthetically.” Peer leader 5: “I think it's a nice looking app.”
	Other barriers	Dislikes technology/ apps	Walker 4: “I’m not an app person.” Walker 8: “I get frustrated quite quickly with that type of technology so I didn’t really bother that much with it…… I spend my whole day on a computer, so I like to minimize my electronic engagement outside that.”
Perceived impact	Fostering goal achievement	Competence	Walker 3: “But I did used to use it [START app] and especially—I found that very important at the beginning because you’ve got to get motivated and that—it did drive me at the beginning ‘cause it helped me get started. So I will give it that credit. It helped me get started.” Walker 10: “occasionally, [you] would get a message about how did you go against goals and review performance and stuff like that. But—which—yeah, was useful just to see—be it on a weekly basis, how the previous week was. I guess I was relatively—oh, I could picture sort of where I was at during the week or at the end of a week as to what I set myself as a goal. So, I think I’ve had a reasonable understanding of how I was going probably necessarily without looking at the summary from the app, but it was still useful to sometimes read through that.” Walker 11: “I think they [motivational messages] made me feel more confident in that I can achieve my goals—encouraging that you can achieve it. Yeah.” Walker 11: “Yeah, they’re [motivational messages] good. They’re good reminders. And it’s always nice to have motivation ‘cause sometimes you sort of—your own mind can say, “Oh, no, not today. I can’t be bothered,” but then to have that, “Oh, yeah, I can do this.” Yeah. Yep. No, they were good.”
		Self-monitoring	Walker 3: “And I guess whilst we were doing the program, entering the data was easy to do because you wanted to see how your other team members were progressing, as in how far we had got to our challenge. So I was always wanting to enter my daily steps.” Walker 10: “I saw the value in having to enter the steps into the START app as sort of acknowledging progress for the day. And it I guess forces you to then see what you—how you’ve ended up against your goal, whereas the temptation might be if you’re not physically doing that each day or every couple of days, then it may be easier to lose sight of how you’re going against the goals. So, I think that worked reasonably well.” Walker 11: “…it was good entering your steps and it was encouraging to—entering your steps using an app ‘cause you think, “Oh, right, 2,000 more steps.” So, it was good.” Peer leader 4: “…it was good to be able to look back and see your progress over the weeks, you were walking in one week as opposed to another week, and what might have been an impact to that week if you didn't do so well.”
	Motivation for walking and other physical activities		Walker 1: “I never really thought much about going for a walk by myself [without the dogs]. But then I started doing it [at work] after the group walks sort of stage stopped. And it was really kind of relaxing. I found it good as well as—obviously, its physical exercise, but it was much more relaxing than I thought it’d be, and sort of helped reset my day in the middle of the day, sort of at lunchtime.” Walker 5: “… now I actually found myself—instead of meeting up with coffee with a friend, actually going for a walk instead.”

Table includes themes, sub-themes, and meaning units from post-trial interviews with walkers and peer leaders, and written comments provided within the uMARS

#### App usage

On average, participants recorded their step counts 4.52 (*SD* = 2.48; range = 0.06–7) times per week. Participants logged an average of 0.68 (*SD* = 1.19; range = 0–4.88) walk activities (i.e., specifying when, where and with whom they would walk) per week on the app. Step count goals were achieved on 2.33 (*SD* = 1.82; range = 0.06–5.81) days per week. Half of participants (25 out of 50 walkers) continued to log steps on at least a weekly basis throughout the intervention.

#### Associations between app usage and behavioral engagement

At the between-person level, participants who entered their step counts on the app more often (frequent *logging* of steps) took more steps (as measured via the Fitbits), than those who engaged less with this function of the app (*b* = 507.60; [95% CI = 240.07–775.12]; *P* < 0.001). Further, at the within-person level, in weeks when participants entered their daily steps often on the app, they accumulated more steps, than during weeks when they entered their daily steps less often (*b* = 181.30; [95% CI = 37.65–324.95]; *P* = 0.016). We also conducted cross-lagged analysis to examine if weekly entering of step counts on the app predicted step counts recorded by the Fitbits the following week at both the between- and within-subject level. The results at the between-subject level showed that participants who entered their step counts on the app more often took more steps (as measured by the Fitbits) in the subsequent week, than participants who logged steps on the app less often (*b* = 503.41; [95% CI = 262.69–744.13]; *P* < 0.001). At the within-person level, during weeks when participants entered their steps on the app often, they accumulated more steps the following week than during weeks when they logged their steps less often (*b* = 166.98; [95% CI = 5.64–328.33]; *P* = 0.04).

There were no between-person differences in step counts between those who logged walking activities more versus less (*b* = 612.06; [95% CI = − 610.78–1834.91]; *P* = 0.27). However, at the within-person level, during weeks when participants logged walking activities more often than usual, they accumulated more steps (*b* = 328.97; [95% CI = 81.91–636.03]; *P* = 0.015). Results of the cross-lagged analysis for this outcome showed no between- (*b* = 592.18; [95% CI = − 567.25–1751.62]; *P* = 0.26) nor within-person level effects (*b* = 141.05; [95% CI = − 179.83–461.93]; *P* = 0.36).

#### Perceived impact in supporting behavioral change

Both walkers and peer leaders rated the app moderately for its perceived impact in supporting behavior change (Table 3), as demonstrated by uMARS scores for perceived impact (*M* = 2.92, *SD* = 1.16; *M* = 3.23, *SD* = 1.10), increase motivation (*M* = 3.06, *SD* = 1.03; *M* = 3.71, *SD* = 1.50), and increase walking behavior (*M* = 2.82, *SD* = 1.36; *M* = 3.57, *SD* = 1.40) for walkers and peer leaders, respectively.

In the interviews, walkers reported that they experienced feelings of achievement (main theme) through building competence and via self-monitoring (sub-themes) from using features of the app including setting step goals and entering daily step counts, and from receiving motivational messages. When walkers were asked specifically about how the weekly motivational messages (delivered using need-supportive communication style) made them feel, they stated feeling more confident in achieving walking goals. Throughout the interviews, feelings of achievement were described as playing an important and influential role in the participants’ decision to increase walking behaviors. Walkers also reported feeling a greater sense of enjoyment and importance (e.g., prioritizing walking), reflecting more self-determined motivation towards walking and other physical activities. Examples of meaning units are presented in Table 4 and Table S1. Peer leaders also perceived the app as useful in supporting goal achievement by being able to review progress over the weeks. Although some walkers reported discontinued use of the app as the program progressed (reasons included not requiring the app as walking becoming part of their routine or being prompted by reminders from the Fitbit), some acknowledged that the app assisted in providing the motivation they required at the beginning of the program.

## Discussion

The purpose of this study was to develop and explore the feasibility of the START app among physically inactive office workers who took part in a 16-week peer-led walking intervention. With regards to acceptability, questionnaire results and interviews with participants indicated that the app was viewed favorably in terms of overall quality, functionality, and aesthetics. Subjective quality and perceived impact, on the other hand, were rated lower (their mean score was closer to or below midpoint) and upon inspection of comments provided by walkers, participants reported a number of factors and barriers to use which may have limited engagement with the START app. First, a small number reported resistance to using the app (i.e., disliking technology or avoided using additional technology), as their occupation required extensive use of a computer. It is important that different preferences (computer-based platforms for entering step counts on work computers or paper-based options) are considered in future studies investigating internet-based technologies among office workers [41]. Second, some walkers reported inconvenience in manually entering their step count, preferring that the START app had the capability to sync with their Fitbit. Manual entry is characteristic of BCT *2.3 self-monitoring* [34] which has been shown to be one of the most effective BCTs in the target population [10]. According to Michie’s taxonomy [34], syncing step counts from the Fitbit to the START app might be considered *2.2 feedback on behavior* or *2.7 feedback on outcomes of behavior*. Along with goal setting and self-monitoring of behaviour, feedback on outcomes of behaviour has been associated with long term intervention success [10], thus it would be beneficial for future work to investigate differences in effective of manual entry (self-monitoring) versus devices that sync with an app then provide opportunities for self-reflection (feedback on outcomes of behavior). Lastly, several walkers reported preferring to use the Fitbit app more than the START app. Participants were provided a Fitbit device one week prior to commencement of the START program (and therefore access to the START app) to measure baseline step count. As participants had access to the Fitbit app prior to the start of the trial and access to the START app, they may have become familiar with using the Fitbit app and, therefore, more likely to be reluctant to using an additional app (i.e. the START app).

Participants demonstrated greater app usage with entering daily step counts than logging walk activities as they happened (i.e., by pressing start/stop buttons at beginning/end of walks). Three factors may explain this difference in the use of the two functions. First, walkers reported perceptions of value and benefit from tracking progress towards their individual step count goal, whereas they did not identify any personal benefits from logging walk activities. Second, contributing to the team destination goal may have created a greater sense of accountability, and therefore motivation, among walkers to enter their daily step count. Finally, some participants reported inconvenience in carrying a device on walks and lack of internet access for recording walk activities (particularly if provided with an iPad), whereas entering step count could be completed at any time. Entering walking activities on the app just before a walk was an exercise to promote practical and emotional social support during the walk (via mid-activity motivational messages), and to monitor emotional consequences (via post-walk reflections) [34]. Clearly, more work is needed to make the use of such an activity more appealing and better understood and valued by the participants.

App usage (entering daily step count and logging walk activities) was positively associated with weekly step count (assessed via Fitbit). These findings are in concordance with previous suggestions that ongoing app usage or engagement (i.e., exposure to the intervention) is important for an intervention to have an effect [42, 43]. However, our study showed only 50% of walkers remained engaged with the app over the full 16-week intervention, aligning with Yardley et al. [24] whom reported that app engagement decline is prominent in smartphone app intervention studies. Although some walkers reported a reduction in app use as the intervention progressed, several commented on motivational messages being useful at the beginning of the program. Walkers (including some that discontinued engagement with the app) reported that certain features of the app, such as motivational messages and ability to record step counts, provided a sense of confidence (competence) and an ability to track or visualize progress (self-monitoring), thereby fostering goal achievement. These results indicate that the BCTs embedded within the app have potential to be effective in assisting behavior change [8–10, 12, 32], even if only for an initial period of engagement. Consequently, some walkers may not have felt it necessary to engage with the app for the full 16 weeks in order to sustain or increase their walking behavior. This finding supports other findings [24], that after an initial period of engagement with a digital intervention, a user may reach sufficient self-regulation, meaning they no longer require the app.

Setting step goals, entering daily step counts, and receiving motivational messages were app features identified by walkers as assisting with promoting walking behavior, it is therefore suggested that BCTs underpinning these app features (goal setting, self-monitoring, and prompts) are considered in future mHealth interventions aimed at increasing physical activity behavior among overweight and obese adults [10, 32] and office workers [12].

Drawing from the principles of SDT [13], BCTs were delivered via a need-supportive communication style (e.g., language used in goal progress messages, mid-walk motivational, and activity reminder messages). In interviews, walkers reported feeling a sense of accomplishment and confidence from using the START app, suggesting that their interactions with the app supported their need for competence [13]. Although this study did not compare the perceived impact of need-supportive communication in promoting walking behavior to other styles of communication (i.e., neutral or need-thwarting [44]), our qualitative results indicate that need-supportive style messages, particularly those that target feelings of competence, may be an important design element for consideration in future mHealth interventions. These findings support previous technology-based studies that recommend the use of autonomy-supportive communication and competence building, to promote behavior change among insufficiently active [22] and overweight and obese adults [10], and office workers [45].

Collectively, questionnaire (perceived impact, increased motivation, and increased walking behavior) and interview data provide support for the app being perceived as effective in improving self-determined motivation towards walking and physical activity. In the interviews, walkers reported feeling a greater sense of enjoyment and importance (e.g., prioritizing walking), reflecting more self-determined motivation [13]. These results indicate that need-supportive motivational messages and reminders may play an important role in developing more autonomous forms of motivation towards walking and physical activity in the context of workplace walking interventions.

### Limitations and future research directions

Although our results indicate that BCTs embedded in the static and dynamics app content may be effective in promoting walking behavior, we did not obtain app usage data for all app features (i.e., our analysis was limited to features relating to reporting physical activity). In the future, it is suggested researchers investigate the dose–response for all features within the app (i.e., which app features participants engage with, how frequently, and for what duration), thereby allowing researchers to identify and quantify the effectiveness of all embedded BCTs in promoting walking behavior. Researchers also may employ Ecological Momentary Assessment methods to explore the immediate (real-time) impact of use of app features such as motivational messages, reminders, and entering step counts upon psychological needs to track within-person behavior change overtime [23, 46].

Acceptability and engagement with the START app may have been influenced by intervention features such as mode of delivery (e.g., app-based and only available on iOS devices), physical environment (e.g., internet access), individual preferences, and prior and concurrent use of the Fitbit app [23]. Additionally, beta-testing was conducted with a physically active sample. Although participants provided useful information regarding functionality and aesthetics which guided the development of the final app, recruiting insufficiently physically active office workers at beta-testing may have allowed for specific feedback relating to improving acceptability and potential engagement among users within the target population. Prior to conducting future RCTs involving mHealth platforms, it is suggested that researchers conduct preliminary exploratory research (including formative and think-aloud interviews) with the target population to better understand, and control for, potential factors influencing engagement with an app [19, 47], and consider theoretical frameworks for enhancing engagement with mobile and digital health interventions [47, 48]. To control for the influence of engagement with other apps (e.g., Fitbit app), it is suggested that future studies use step count devices that do not require installation of an additional app or mHealth platforms. Alternatively, researchers may choose to partner with commercial app developers to incorporate evidence-based BCTs and need-supportive communication into existing apps aimed at promoting physical activity among insufficiently active adults.

Although we investigated perceived impact of the app in supporting behavior change via the uMARS, engagement data, and interviews, we did not directly assess changes in motivation for walking. The Behavioral Regulation for Walking Questionnaire [49] was used in the larger trial [25], however this trial also included need-supportive communication from peer leaders and the results, therefore, cannot be solely attributed to the use of the app. To understand the independent effects of need-support provided from the app and from peer leaders, it is recommended that future RCTs are designed with multiple groups to isolate the relative effects of these conditions (i.e., control, START app only, peer-leaders only, and both), and with an adequate sample size to have sufficient power [50]. Evidence for adopting a multi-armed design is supported by recommendations following development and evaluation of the *Active Coach* app [19, 51]. Based on BCTs and autonomy-supportive communication style, the authors found that the app was deemed acceptable and feasible among the target population [19] yet the app alone was not effective promoting physical activity levels [51], with recommendations for future studies to include multicomponent interventions including both app and human support. Further, it is recommended researchers consider incorporating *Motivation and Behavior Change Techniques* (MBCTs [52]) and the *Taxonomy of App Features Based on SDT* [18] in future app development.

## Conclusions

In conclusion, we showed that the START app may be a useful mHealth tool for promoting walking behavior among insufficiently active office workers, as it was rated favorably in terms of acceptability by walkers and peer leaders, app usage was positively associated with weekly step count, and it targeted specific evidence-based means (BCTs based on a need-supportive style of communication) to support participants’ self-determined motivation for behavior change. Results from this study can be used to inform the development of future apps specifically targeting insufficiently active overweight and obese office workers to increase walking.

## Supplementary Information

**Additional file 1**. CONSORT checklist.

**Additional file 2**. Virtual walk categories, post-trial interview guides, and full thematic analysis.

## Data Availability

The datasets generated and/or analyzed during the current study are available from the corresponding author on reasonable request.

## Abbreviations

BCTs: Behavior change techniques
BMI: Body mass index
MVPA: Moderate to vigorous physical activity
RCT: Randomized controlled trial
START: Striding TowARds health and well-being Trial
SDT: Self-Determination Theory
