# Supplementary material for: Development and feasibility of a mobile phone application designed to support physically inactive employees to increase walking
Source: BMC Med Inform Decis Mak. 2021 Jan 21;21:23. doi: 10.1186/s12911-021-01391-3 (PMC7819207; doi:10.1186/s12911-021-01391-3)
Supplement: Supplementary file 2 — Additional file 2. Virtual walk categories, post-trial interview guides, and full thematic analysis. [file 12911_2021_1391_MOESM2_ESM.docx]

**Supplementary material**

**Virtual walks categories**

Virtual walks were categorized into easy, medium and hard difficulty level based on the below calculations:

- Easy (40,000 to <52,500 steps per week per participant)

5000 steps per day on 5 days of the week = 25,000

+ 7500 steps per day on 2 days/wk = 15,000

Total = 40,000

- Medium (52,500 to <60,000 steps per week per participant)

7,500 steps per day on all days of the week = 52,500

Total = 52,500

- Hard (60,000 to 70,000 steps per week per participant)

10,000 steps per day on 3 days per week = 30,000

+ 7,500 steps on 4 days per week = 30,000

Total = 60,000

For groups where the majority of participants had a low baseline step count (e.g., <3,000 steps a day) and/or had members who found it difficult to walk at a moderate pace for 20 min, the peer leader selected a challenge destination from the ‘easy’ category.

**Post-Trial Interview Guide for Walkers.**

- Can you tell me about your experiences of using the START app? Prompts: What did you like about the app? What didn’t you like about the app?

*Prompts: functions, ease of use, aesthetics, information*

- Did you choose to use some functions and not others? If so why? Functions include: entering daily steps, adjusting step goal, walking reminders, recording walks.

*Prompt if indicating they didn’t use it much: what were some reasons or barriers for not using the app daily/more often (e.g. entering step count each day)?*

- What did you think about the weekly motivational messages? How did they make you feel?

*Prompts: how did they make you feel about:*

- - *Your ability to meet your goals?*
  - *Your perceptions of feeling in control of your walking schedule?*
  - *Your perceptions of feeling valued and cared for?*

**Post-Trial Interview Guide for Peer Leaders.**

- Can you tell me about your experiences of using the START app? What did you like about the app? What didn’t you like about the app?

*Prompts: functions, ease of use, aesthetics, information*

- - If peer leader indicates that they didn’t use the app much ask - What were some reasons or barriers for not using the app daily/more often?
- Did the START app help you in your role as a peer leader? How?

**Table S1.** Acceptability and perceived impact of the START app in supporting behavior change.

|  | Theme | Sub-theme | Exemplar meaning unit |
| --- | --- | --- | --- |
| Acceptability | Functionality | Ease of use | Walker 1: “I found it pretty easy to use. I never had any problems with it. It was pretty intuitive and – yeah, it was pretty basic and effective, easy to read and understand.”  Walker 5: “I actually found it very easy to work, to use, and – yeah, I thought it was just really basic and really just – I mean, it did what it had to do and easy to use.”  Walker 6: “I was easily able to input the steps every day and put some reminders in my phone and stuff.”  Peer leader 3: “Yep. I used it for every walk and I found it really good, a really good way of recording the walks and it worked really well, so I would just insert the basic details, who’s going on the walk, what type of walk, and then go press start and stop. We pretty much used it all the time.”  Peer leader 4: “It was easy to use, and you could also edit. You started to walk and you didn't have time to put in who was walking or where you were walking, once you got back, you could edit just to add the information in where you’d finished the walk”.  Peer leader 5: “So I found that it was pretty easy to use. I didn’t really have any problems with it. Once it was set up on my phone, logging people in to go walking, I found it easy”. |
|  |  | Manually entering daily step count and influence of Fitbit app | Walker 3: “I would only just say you could sync it in [with the FitBit]. That would be the only thing ‘cause we just live in a world where – we’re just so fast. Everything’s done for us. A bit lazy, I know.”  Walker 6: “I probably did not use it to its full capacity. Just entered activity and steps each day.”  Walker 11: “I love the little [START] app that you can enter your steps.”  Walker 10: “I found the app to be really just an entry portal for data for the purpose of visibility for the Curtin START team. Predominantly I used the Fitbit app as the main source, and then just entered the step data into the START app.”  Walker 13: “Would be great if it linked directly with Fitbit. I failed to record my steps on days when I accidently turned bluetooth off. Also, when I first started walking [I] would enter my steps every day then as the days went by I would.”  Walker 14: “We needed to engage with the Fitbit app to interact with the START App, which made the START app redundant to the Fitbit app. If the Fitbit app automatically sent steps into the app, it would have been easier to engage with the app.” |
|  |  | Limited to Apple devices | Walker 7: “So I had it [START app] on my iPad and a lot of the other people had them on their phones which might – I realised now, but I wasn’t at that time sure I could do that. So they can actually use it to track what they were doing, how they were feeling, and who they were with. I had to do mine when I got back in the evening. So it didn’t track my progress at all.”  Walker 9: “… if it was designed for both Android and Apple, you’d be extremely successful at it.”  Walker 9: “So I didn’t use most of those functions because I didn’t have an iPhone. So, I had the iPad and if I went out walking, there was no point in giving me inspiration halfway around, whereas if I had the phone, it would’ve been a lot more useful, I think. But I just – yeah, I had it on and then I thought, “Well, there’s no point at all.” So I turned it off I think in the first week or so, those functions.”  Walker 14: “I also had to use an iPad as I did not have an iPhone making the app more inconvenient as I needed to be connected to wifi, which I did not have access to at work.” |
|  | Aesthetics |  | Walker 6: “Yeah. Yeah, it was fine.”  Walker 9: “It’s a bit plain, to be honest, aesthetically.”  Peer leader 1: “… the interface was fine.”  Peer leader 5: “I think it's a nice looking app.” |
|  | Other barriers | Dislikes technology/ apps | Walker 4: “I’m not an app person.”  Walker 8: “I get frustrated quite quickly with that type of technology so I didn’t really bother that much with it…... I spend my whole day on a computer, so I like to minimize my electronic engagement outside that.” |
| Perceived  impact | Fostering goal achievement | Competence | Walker 3: “But I did used to use it [START app] and especially – I found that very important at the beginning because you’ve got to get motivated and that – it did drive me at the beginning ‘cause it helped me get started. So I will give it that credit. It helped me get started.”  Walker 10: “occasionally, [you] would get a message about how did you go against goals and review performance and stuff like that. But – which – yeah, was useful just to see – be it on a weekly basis, how the previous week was. I guess I was relatively – oh, I could picture sort of where I was at during the week or at the end of a week as to what I set myself as a goal. So, I think I’ve had a reasonable understanding of how I was going probably necessarily without looking at the summary from the app, but it was still useful to sometimes read through that.”  Walker 11: “I think they [motivational messages] made me feel more confident in that I can achieve my goals – encouraging that you can achieve it. Yeah.”  Walker 11: “Yeah, they’re [motivational messages] good. They’re good reminders. And it’s always nice to have motivation ‘cause sometimes you sort of – your own mind can say, “Oh, no, not today. I can’t be bothered,” but then to have that, “Oh, yeah, I can do this.” Yeah. Yep. No, they were good.” |
|  |  | Self-monitoring | Walker 3: “And I guess whilst we were doing the program, entering the data was easy to do because you wanted to see how your other team members were progressing, as in how far we had got to our challenge. So I was always wanting to enter my daily steps.”  Walker 10: “I saw the value in having to enter the steps into the START app as sort of acknowledging progress for the day. And it I guess forces you to then see what you – how you’ve ended up against your goal, whereas the temptation might be if you’re not physically doing that each day or every couple of days, then it may be easier to lose sight of how you’re going against the goals. So, I think that worked reasonably well.”  Walker 10: “I guess so. Yeah, I guess so, in – well, as I said, I think I felt – well, I knew when I was in control – I think on one occasion, I had a bit of an injury. So, I knew I was gonna be a lot less active for that week, but – yeah, having known that, it was a case of – I didn’t expect it by the end of the week that I was gonna be on track. But for the rest – most of the rest part of – I guess had a reasonable picture of where I was. But the app is to obviously help visualize that, you know?”  Walker 10: “I think having the target set – and although I didn’t update as I updated the target, I think I had mentally certainly had a target in my head and I was – because I was still entering in the steps each day, I knew whether I was meeting that target or not. So, the fact that it later stages wasn’t showing up against the updated target, I think it wasn’t – didn’t seem to affect how – or what I was getting out of it.”  Walker 11: “…it was good entering your steps and it was encouraging to – entering your steps using an app ‘cause you think, “Oh, right, 2,000 more steps.” So, it was good.”  Peer leader 4: “…it was good to be able to look back and see your progress over the weeks, you were walking in one week as opposed to another week, and what might have been an impact to that week if you didn't do so well.” |
|  | Motivation for walking and other physical activities |  | Walker 1: “I never really thought much about going for a walk by myself [without the dogs]. But then I started doing it [at work] after the group walks sort of stage stopped. And it was really kind of relaxing. I found it good as well as – obviously, it’s physical exercise, but it was much more relaxing than I thought it’d be, and sort of helped reset my day in the middle of the day, sort of at lunchtime.”  Walker 3: “I like it [being active] now. I am – I’m in tune. I’m enjoying the gym. Me, the last person in the world – I actually won a 12-week membership which made me – and I walked in to that gym and I went, “You know what? I’m gonna do this 12 weeks and if I can do this 12 weeks, I’m staying.” And I’m staying. It’s not gonna take me 12 weeks to make that decision. I’m staying. I’m enjoying. I’m actually enjoying [it] and work has a corporate membership. I never even knew that. But then again, I couldn’t have cared less about exercise before, so that – it may have been that I just wasn’t in that frame of mind, whereas I’m in that frame [of mind] now where I don’t care about – the weight is not an issue. I’m genetically gonna be overweight. That’s the way it is in our family unfortunately, but I’m actually enjoying being that little bit healthier.”  Walker 5: “Yeah. I’m still walking. I probably dropped off the walking a little bit ‘cause I’ve taken up yoga. So – but I’ve also started – towards the end of the program, I started doing geocaching with a friend, which is a walking thing as well. So I think I tend to do things like that to kind of keep my interest. So, for me, something like geocaching or orienteering, which I am planning on doing the metro series on orienteering, so just walking around but not running. So, just that’s – it’s just something else that’s there to make it more interesting. And in the past, I’ve also done the Nordic walking, so I have Nordic walking poles, so I was thinking about going back into that as well. So, yeah, it’s – the program has certainly – yeah, just motivated me to continue.”  Walker 5: “... now I actually found myself – instead of meeting up with coffee with a friend, actually going for a walk instead.”  Walker 10: “I guess it’s helped steer a bit of focus. I’ve not ever really disliked walking. It’s just – I’ve not, I guess, dedicated the time sufficiently to do that. And I think the program has helped me to sort of get into a bit of a routine and I do very much enjoy walking, and even jogging now. So, I think it has helped just with having the discipline and knowing that you can do these things without – and still go back doing all the other things you need to do during the day. So, that’s been useful.”  Walker 11: “I just wish <laughs> – this morning I was meant to get up at 5:30 and go for a walk, but bed looked too good. But – yes, I do – I really want to set time aside – at least a few times a week to just go for a walk. That’s – ‘cause it’s shown me how much I do enjoy walking and just how much you see when you go for a walk.” |

Note: Table includes themes, sub-themes, and meaning units from post-trial interviews with walkers and peer leaders, and written comments provided within the uMARS.
